# Supplementary figures and images for: Simultaneous Tracking of Pseudomonas aeruginosa Motility in Liquid and at the Solid-Liquid Interface Reveals Differential Roles for the Flagellar Stators
Source: mSystems. 2019 Sep 24;4(5):e00390-19. doi: 10.1128/mSystems.00390-19 (PMC6759568; doi:10.1128/mSystems.00390-19)

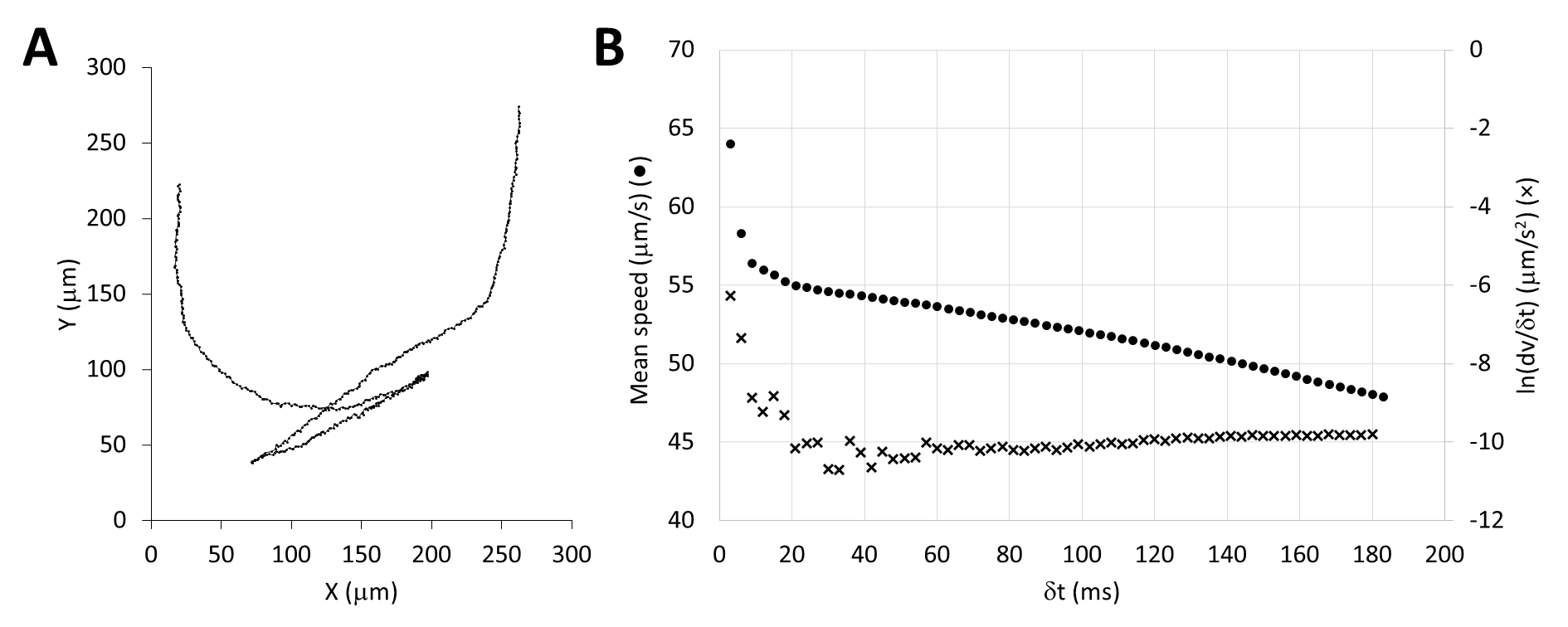

Supplement: FIG S1 [file mSystems.00390-19-sf001.tif]

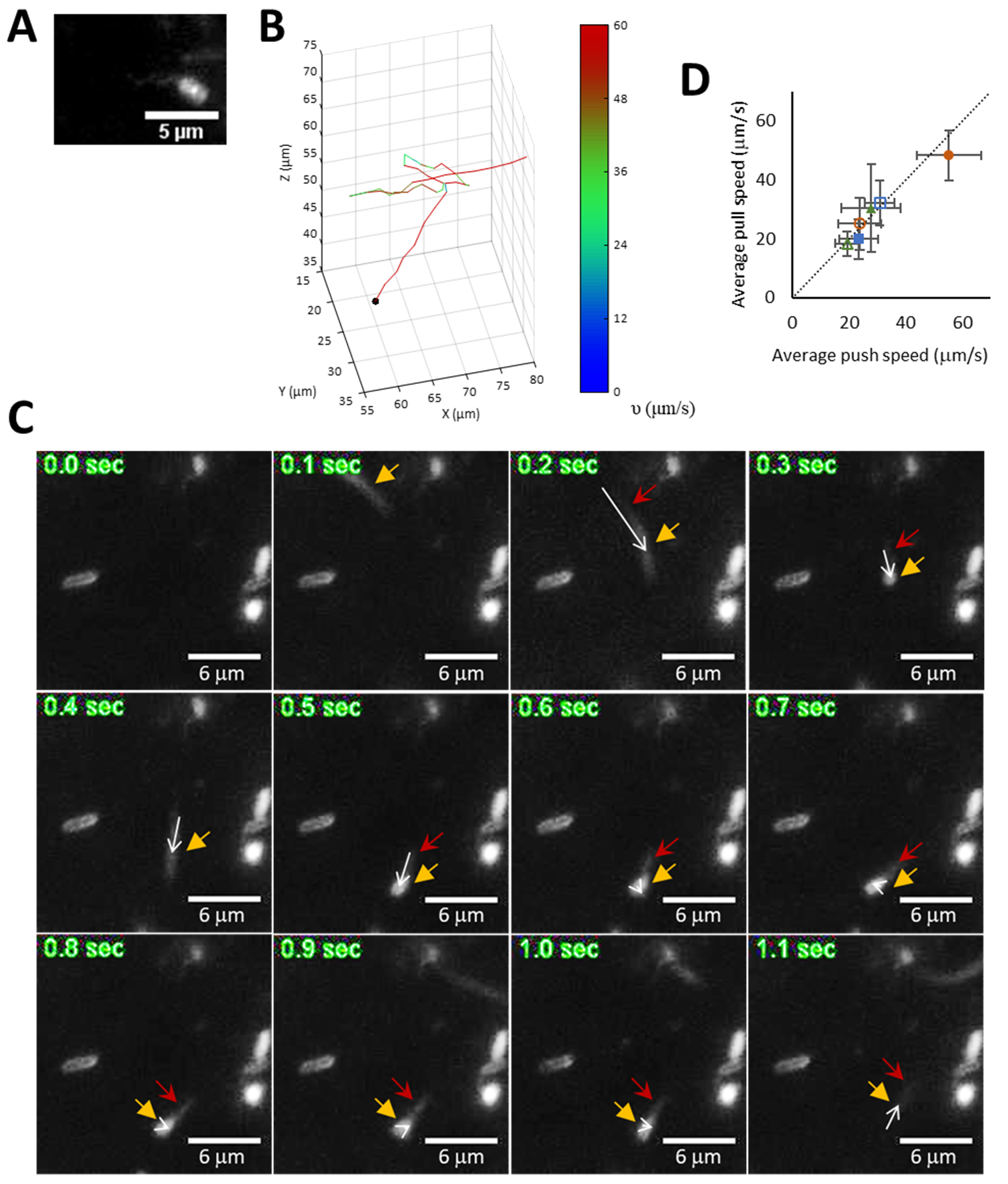

Supplement: FIG S2 [file mSystems.00390-19-sf002.tif]

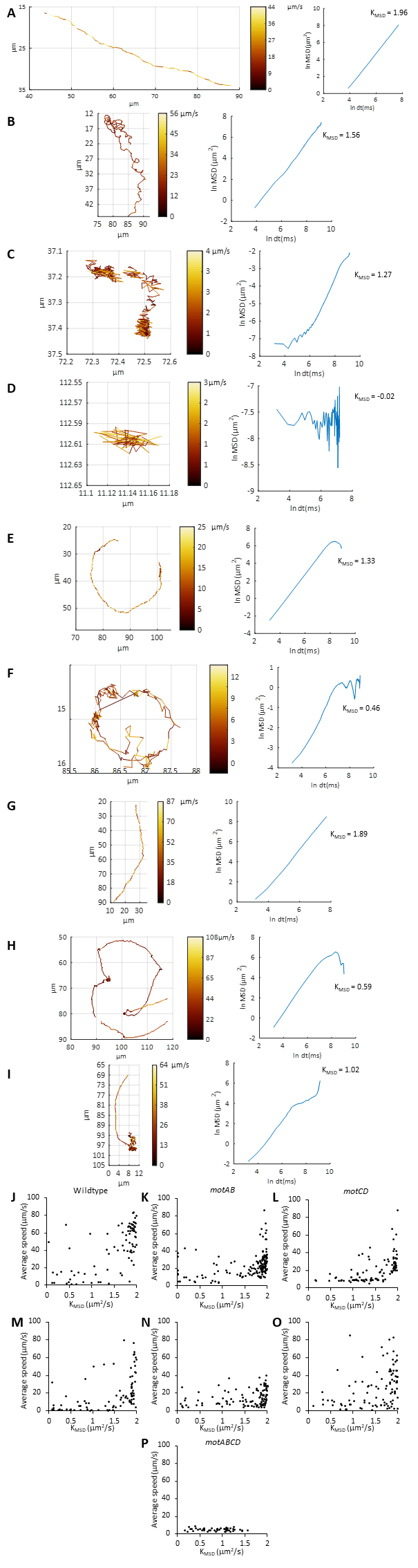

Supplement: FIG S3 [file mSystems.00390-19-sf003.tif]
