# Supplementary material for: Simultaneous Tracking of Pseudomonas aeruginosa Motility in Liquid and at the Solid-Liquid Interface Reveals Differential Roles for the Flagellar Stators
Source: mSystems. 2019 Sep 24;4(5):e00390-19. doi: 10.1128/mSystems.00390-19 (PMC6759568; doi:10.1128/mSystems.00390-19)
Supplement: TABLE S1 [file mSystems.00390-19-st001.pdf]

Name	Sequences
**MotABΔ FW1**	5’-ATATCTAGAAGTTGGCTCGCGCGTCCCAG-3’
**MotABΔ RV1**	5’-TCAGTCGCCCTTGATGATTTTTGACATGAGGACCGG-3’
**MotABΔ FW2**	5’-CTCATGTCAAAAATCATCAAGGGCGACTGAGGTTCA-3’
**MotABΔ RV2**	5’-TATAAGCTTTGGTGAATGCCTTGTTGCCA-3’
**MotCDΔ FW1**	5’-ATATCTAGAAGCAGATGATGGTCGACGGC-3’
**MotCDΔ RV1**	5’-TCATGGCGAAGGCGAGAGCACATCCATCAGCGCGTC-3’
**MotCDΔ FW2**	5’-CTGATGGATGTGCTCTCGCCTTCGCCATGAGCGCGC-3’
**MotCDΔ RV2**	5’-TATAAGCTTAGGCTCTTCGAGACCACCAG-3’
**MotAB FW**	5’-TATGAATTCATGTCAAAAATCATCGGC-3’
**MotAB RW**	5’-TATCTCGAGTCAGTCGCCCTTGATCTG-3’
**MotCD FW**	5’-TATGAATTCATGGATGTGCTCAGCCTG-3’
**MotCD RW**	5’-TATCTCGAGTCATGGCGAAGGCGACGG-3’
